# *Anopheles gambiae* s.l (Diptera: Culicidae) seasonal abundance, abdominal status and parity rates in Metema-Armachiho lowland, Northwest Ethiopia

**DOI:** 10.1186/s12879-020-05068-6

**Published:** 2020-05-11

**Authors:** Yibeltal Aschale, Animen Ayehu, Ligabaw Worku, Ayenew Addisu, Ayalew Jejaw Zeleke, Abebe Genetu Bayih, Wossenseged Lemma

**Affiliations:** 1 Department of Medical Parasitology, College of Health Sciences, Debre Markos University, P.O. Box: 269, Debre Markos, Ethiopia; 2 Department of Medical Parasitology, College of Medicine and Health Sciences, Bahir Dar University, Bahir Dar, Ethiopia; 3 Department of Medical Parasitology, School of Biomedical and Laboratory Sciences, College of Medicine and Health Sciences, University of Gondar, Gondar, Ethiopia; 4 Director General,Armauer Hansen Research Institute (AHRI), Addis Ababa, Ethiopia

**Keywords:** Seasonal abundance, *An.gambiae s.l*, Abdominal status, Parity

## Abstract

**Background:**

Malaria is a life threating vector borne disease caused by different *Plasmodium* parasites. Metema and Armachiho are two of the top five malaria endemic areas among the districts of Amhara region in Ethiopia. Transmission pattern is seasonal and migrant laborers who visit these areas for employment in mechanized agriculture are highly affected. The aim of this study was to investigate seasonal abundance, abdominal status and parity rate of *An.gambiae* s.l in Metema-Armachiho lowlands, Northwest Ethiopia.

**Method:**

A 1 year longitudinal entomological study was conducted in Metema-Armachiho lowlands from June 2016–May 2017. Mosquitoes were collected using CDC-light traps in indoor and outdoor sites for four consecutive days in each month. A total of eight standard battery operated CDC-light traps were used to collect mosquitoes. Female mosquitoes were classed as unfed, fed or gravid under a dissecting microscope. The ovaries of all unfed *An.gambiae* s.l mosquitoes were examined for evidence of parity. Data were entered and analyzed using SPSS-20 software. Chi-square test was applied to show significant difference between variables. *P*-value< 0.05 was taken as statistically significant.

**Results:**

Of the total 1253 mosquitoes collected, 713 (552 female, 161 male) were *culex* and 540 (501 female, 39 male) were *An.gambiae* s.l. About 50.9% were collected in June–August 2016, 21.7% in September–November 2016, 12.0% in December 2016–February 2017 and 15.4% in March to May 2017. Of the total, 57.2 and 42.8% of the *An.gambiae* s.l were collected from indoor and outdoor sites respectively. Of the total females collected, 76.8% were unfed; of which 69.4% were parous. Significantly higher number of female *An.gambiae s.l* were collected in indoor and there was significant difference in abdominal status of *An.gambiae s.l* mosquitoes collected in different season (*P* < 0.05).

**Conclusions:**

Highest number of *An.gambiae* s.l was observed from June–August followed by September–November. The parity rate of *An.gambiae* s.l was high and there was significant difference in abdominal status of *An.gambiae s.l* collected in different season.

## Background

Malaria burden is high in poor and malnourished populations [1]. Labor migrants who travel to endemic areas, under five children and pregnant women are high risk groups for malaria infection [2–4]. Malaria transmission in Ethiopia is seasonal and affected by rainfall and altitude patterns [5–7]. Transmission period varies from a few weeks prior to the main rainy season (April–June) to more than a month following main rainy season (September–December) which coincides with the major harvesting and planting seasons [5, 8, 9]. Thus, it has a severe impact on Ethiopia’s economy by cutting down productivity capacity [10].

Family Culicidae consists of two principal lineages that are recognized as subfamilies, the *Anophelinae* and *Culicinae* [11]. Of the 465 properly identified *Anopheles* mosquito species worldwide, 70 have the ability to transmit malaria parasites in humans [12]. *An. arabiensis,* which belongs to *An.gambiae s.l,* is the primary vector of malaria parasite in Ethiopia. However, *An. pharoensis, An. nili* and *An. funestus* are secondary malaria vectors [13–15]. A large decrease of the important endophilic-endophagic malaria vectors (*An. gambiae* sensu stricto and *An. funestus)* and persistence of the more exophilic-exophagic species (*An. arabiensis)* have been observed now a days [15, 16].

Regular indoor residual spraying (IRS) and long lasting insecticide treated nets (LLITNs) are currently vector control intervention strategies in Metema and Armachiho [5, 7]. Environmental management is also key intervention measure applied to control malaria in the study areas [13, 17]. Modification of the environment has great effect on breeding of mosquitoes. It affects mosquito abundance by altering both quality and quantity of breeding sites [18].

Effectiveness of malaria prevention with LLITNs and IRS is inadequate due to emergence of insecticide resistance, outdoor and early evening/morning biting mosquitoes. New vector control tools are required and their efficiency should be improved. Therefore, studying *An. gambiae s.l* mosquito abundance, abdominal status and parity rate in Metema and Armachiho lowlands, which are among the hot spot areas of malaria in Ethiopia, is essential to design and apply complementary vector control tools that target outdoor and early morning/evening bitters to scale up malaria prevention and to reduce the burden of malaria in the study area.

## Methods

### Study area

The study was conducted in Metema-Armachiho lowlands, Northwest Ethiopia, considered as single survey domain, in three randomly selected villages; Kumer (13°00′N/36°15′E), Selam ber (13°45′N/36°24′E) and Abderafi (13°41′N/36°28′E). The altitude of Metema and West Armachiho Districts is 685 and 667 m above sea level respectively. The minimum annual temperature for both districts ranges from 22 °C to 28 °C. The daytime temperature is very high during March to May (reaches up to 43 °C). These districts are hyper-endemic malarious areas and the weather condition is appropriate for mosquito vector breeding [19].

### Study design and period

A 1 year longitudinal entomological study was conducted from June 2016 to May 2017 in Metema-Armachiho lowlands, Northwest Ethiopia. Entomological surveys were conducted in three villages (four houses from each village) using eight CDC-light traps in indoor and outdoor.

### Mosquito collection and processing methods

#### Mosquito collections

Mosquitoes were collected daily according to the WHO standard protocol using standard battery operated CDC-light traps set overnight (18,00–06:00 h) [20, 21]. A total of 12 houses, four houses from each village, were selected for the survey. Collection was made from 12 houses for four consecutive days in each month. A total of eight CDC-light traps were used to collect mosquitoes indoor and outdoor at 1–2 km distance from potential breeding sites. They were hanged 1.5 m above the ground. Indoor collection was performed in bedroom and outdoor collection was performed in verandas.

#### *Anopheles gambiae* s.l mosquito processing

All collected adult *An. gambiae s.l* mosquitoes were first anesthetized with ethyl ether for 30 min in an anesthetizing chamber. The sex and species of all collected mosquitoes were identified using standard keys [22–24] and labeled according to date and site of collection. Female mosquitoes were classed as unfed, fed or gravid under a dissecting microscope.

#### Dissection and examination of ovaries

The ovaries of all unfed *An.gambiae* s.l mosquitoes were examined for evidence of parity by observing whether they contained eggs or whether the tracheolar skeins surrounding the ovaries were coiled or uncoiled [20–22].

### Data analysis and interpretation

The data was appropriately coded, entered, checked for completeness and analyzed using SPSS 20 software. Descriptive statistics, frequency and cross tabulations were used. The findings were presented in text, table and graph. Chi-square test was used for statistical analysis to show significant difference between variables. *P*-value< 0.05 was taken as statistically significant.

## Result

During the survey a total of 1253 (713 culex, 540 An. *gambiae s.l*) mosquitoes were collected from 192 CDC-light traps in both indoor and outdoor sites. Of the total *An.gambiae s.l samples*, 92.8% (95% CI: 90.5–94.8) were female, and 57.2% (95% CI: 52.9–61.6) and 42.8% (95% CI: 38.4–47.1) were from indoor and outdoor sites respectively. Significantly higher number of female *An.gambiae s.l* mosquitoes were collected in indoor (χ2 = 23.1, *P* < 0.05) **(**Table 1**).**

**Table 1:** Number of *An.gambiae s.l* collected indoor and outdoor between June 2016 and May 2017 in Metema-West Armachiho lowlands, Northwest Ethiopia (*N* = 540)

Site of collection	Sex		Total	χ2 Value	P-Value
	Male *An. gambiae s.l*	Female *An.gambiae s.l*			
Indoor	8 (20.5)	301 (60.1)	309 (57.2)	23.1	< 0.05
Outdoor	31 (79.5)	200 (39.9)	231 (42.8)		
Total	39 (7.2)	501 (92.8)	540 (100)		

### Seasonal abundance of *An. gambiae s.l* mosquitoes

About 50.9% were collected in June–August 2016, 21.7% in September–November 2016, 12.0% in December 2016–February 2017 and 15.4% in March to May 2017. There was significant difference in number of *An.gambiae s.l* mosquitoes collected and season (χ2 = 41.9, *P* < 0.05). In Abderafi, highest number of *An.gambiae s.l* mosquitoes were collected in September–November 2016 and lowest number of *An.gambiae s.l* mosquitoes were collected in December 2016–February 2017. In Selam Ber, highest number of *An.gambiae s.l* mosquitoes were collected in June–August 2016 and lowest number of *An.gambiae s.l* mosquitoes were collected in March–May 2017. Significantly highest number of *An.gambiae s.l* mosquitoes were collected in June to August in Kumer and Selam ber, whereas, significantly highest number of *An.gambiae s.l* mosquitoes were collected in September–November in Abderafi (χ2 = 135.9, *P* < 0.05) (Table 2)**.**

**Table 2:** Seasonal abundance of *An.gambiae s.l* mosquitoes in different villages collected between June 2016 and May 2017 in Metema-Armachiho Lowlands (*N* = 540)

Season	Villages			Total *n* (%)	χ2 value	*P*-Value
	Kumer *n* (%)	Abderafi *n* (%)	Selam Ber *n* (%)			
Jun-Aug 2016	145 (61.7)	45 (24.7)	85 (69.1)	275 (50.9)		
Sep-Nov 2016	15 (6.4)	88 (48.4)	14 (11.4)	117 (21.7)	135.9	< 0.05^*^
Dec 2016-Feb 2017	29 (12.3)	23 (12.6)	13 (10.6)	65 (12.0)		
Mar-May 2017	46 (19.6)	26 (14.3)	11 (8.9)	83 (15.4)		
Total	235 (100)	182 (100)	123 (100)	540 (100)		

*Statistically significant

### Indoor-outdoor distribution of mosquitoes across villages

**Fig. 1:**
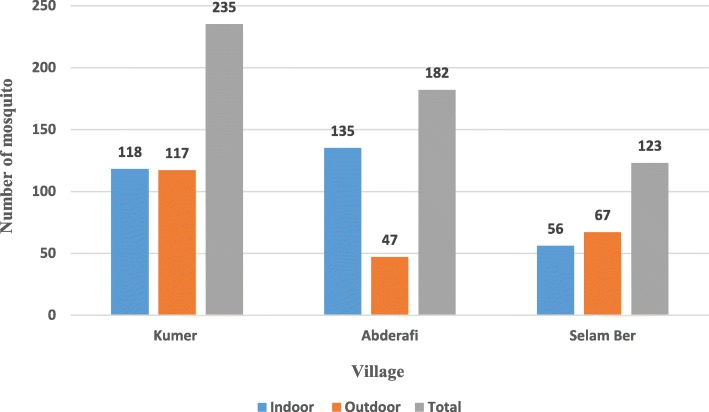
Indoor-outdoor distribution of mosquitoes across villages

In Kumer, approximately equal number of mosquitoes were collected in indoor and outdoor. In Abderafi, higher number of mosquitoes were collected in indoor than outdoor whereas, in Selam Ber there is a slight increase in outdoor. Significantly highest number (235) of mosquitoes were collected from Kumer (χ2 = 32.9; *P* < 0.05) (Fig. 1).

### Abdominal status of *An.gambiae s.l* mosquitoes

The abdomen of 501 female *An.gambiae s.l* mosquitoes was examined to determine abdominal status. Of which, 76.8% (95% CI: 73.1–80.4), 14.4% (95% CI: 11.4–17.4), 8.8% (95% CI: 6.4–11.2) were found unfed, fed and gravid, respectively. There was significant difference in abdominal status of *An.gambiae s.l* mosquitoes collected in different season. Significantly higher number of unfed *An.gambiae s.l* mosquitoes were captured from December to February (*P* < 0.05) (Table 3).

**Table 3:** Abdominal status of *An.gambiae s.l* mosquitoes collected between June 2016 and May 2017 in Metema-West Armachiho lowlands, Northwest Ethiopia (*N* = 501)

Season	Abdominal status			Total *n* (%)	χ2 value	*P*-Value
	Unfed *n* (%)	Fed *n* (%)	Gravid *n* (%)			
Jun-Aug 2016	197 (72.4)	49 (18.0)	26 (9.6)	272 (100)		
Sep-Nov 2016	80 (83.3)	10 (10.4)	6 (6.3)	96 (100)	43.7	< 0.05^*^
Dec 2016-Feb 2017	61 (100)	0 (0)	0 (0)	61 (100)		
Mar-May 2017	47 (65.3)	13 (18.0)	12 (16.7)	72 (100)		
Total	385 (76.8)	72 (14.4)	44 (8.8)	501 (100)		

*Statistically significant

### Parity rate of *An.gambiae s.l* mosquitoes

The ovaries of 385 unfed *An.gambiae s.l* mosquitoes were examined to determine parous rate. Of which, 69.4% (95% CI: 64.9–73.9) *An.gambiae s.l* mosquitoes were found parous and 30.6% (95% CI: 26.1–35.1) were found nulliparous. There was no significant difference in parity rate of *An.gambiae s.l* mosquitoes collected in different season (χ2 = 0.062, *P* = 0.99) (Table 4).

**Table 4:** Parity rate of *An.gambiae s.l* mosquitoes collected between June 2016 and May 2017 in Metema-West Armachiho lowlands, Northwest Ethiopia (*N* = 385)

Season	Parity status		Total *n* (%)	χ2 Value	*P*-Value
	Parous *n* (%)	Nulliparous *n* (%)			
Jun-Aug 2016	137 (69.5)	60 (30.5)	197 (100)		
Sep-Nov 2016	56 (70)	24 (30)	80 (100)	0.062	0.99
Dec 2016-Feb 2017	42 (68.9)	19 (31.1)	61 (100)		
Mar-May 2017	32 (68.0)	15 (32)	47 (100)		
Total	267 (69.4)	118 (30.6)	385 (100)		

## Discussion

This entomological study was the first time study carried out in Northwest Ethiopia to assess *An. gambiae* s.l. seasonal abundance, abdominal status and parity rate. Molecular analysis of *An.gambiae s.l* mosquitoes revealed that *An. arabiensis* is the only vector member of the complex in malaria endemic areas of Ethiopia [14, 25, 26]. Larval ecology indicates that *An. gambiae s.l* larvae develop in freshwater habitats that are small, temporary, clean and sun-exposed. The larva may breed in any available water, even in dirty and polluted habitats [27].

In this study, the highest number of *An.gambiae s.l.* were collected during rainy season of which 57.2% were from indoor sites and 42.8% were from outdoor sites indicating the outdoor host seeking and resting behavior of *An.gambiae s.l*. Significantly higher number of male *An.gambiae s.l* were collected in outdoor and significantly higher number of female *An. gambiae s.l* were collected in indoor (*P* < 0.05). This indicates that only female mosquitoes seek blood for the development of their egg and male normally can survive by feeding on nectar juice. This is comparable with a previous study conducted in Sille, South Ethiopia which demonstrated that 54.2 and 45.8% of *An.gambiae s.l* were collected from indoor and outdoor sites respectively [28]. On the other hand, this finding is somewhat different from a previous study conducted in other part of Ethiopia, in which 44.4% of *An.gambiae s*.l were captured from indoor and 55.6% were collected from outdoor [29]. The reason for different proportions of indoor or outdoor biting might be due to human behaviour. In the study area (Metema and Armachiho district) migrant laborers often work outdoor and sleep in the open field exposing themselves for outdoor biters. This affects host seeking behaviors of *An.gambiae s*.l.

Of all female *An.gambiae s.l* mosquitoes examined for abdominal status, 76.8% were found unfed, 14.4% were found fed and, 8.8% were found gravid. The gravid *An.gambiae s.l* mosquitoes collected outdoor were either coming out to seek resting habitat or they were initially unfed indicating their outdoor resting and biting (partial exophillic-exophagic) behavior. There was significant difference in abdominal status of *An.gambiae s.l* mosquitoes collected in different seasons (*P* < 0.05).

Of all unfed female *An.gambiae s.l* mosquitoes examined, 69.4% were found parous and 30.6% were nulliparous. There was no significant difference in parity rate of *An.gambiae s.l* mosquitoes collected in different seasons (*P* > 0.05). This finding is comparable with previous study conducted in Sille, South Ethiopia with an overall parous rate of 73.2% [28] and higher than a study conducted in East Wellega Zone, West Ethiopia with an overall *An. arabiensis* parous rate of 45% [14]. The reason for this difference might be due to difference in mosquito sampling season, mosquito sampling duration and host availability.

## Conclusions

*An.gambiae* s.l abundance was highest in June–August followed by September–November. The parity rate of *An.gambiae* s.l was high and there was significant difference in abdominal status of *An.gambiae s.l* collected in different season. Supplementary vector control tools like larval source management and use of vapor phase repellents are required to tackle outdoor or early biting. In addition, there should be active surveillance including asymptomatic carriers.

## Data Availability

The datasets used and/or analyzed during the current study are available from the corresponding author upon reasonable request.

## Abbreviations

IRS: Indoor Residual Spray
LLITNs: Long Lasting Insecticide Treated Nets
s.l: sensu lato
